# Genomic vulnerability of a freshwater salmonid under climate change

**DOI:** 10.1111/eva.13602

**Published:** 2023-10-27

**Authors:** Anna Tigano, Tyler Weir, Hillary G. M. Ward, Marika Kirstin Gale, Carmen M. Wong, Erika J. Eliason, Kristina M. Miller, Scott G. Hinch, Michael A. Russello

**Affiliations:** ^1^ Department of Biology The University of British Columbia Kelowna British Columbia Canada; ^2^ Fish and Wildlife Branch British Columbia Ministry of Forests Victoria British Columbia Canada; ^3^ Resource Management British Columbia Ministry of Forests Penticton British Columbia Canada; ^4^ Freshwater Fisheries Society of BC Victoria British Columbia Canada; ^5^ Yukon Field Unit Parks Canada Whitehorse Yukon Territories Canada; ^6^ Department of Ecology, Evolution, and Marine Biology University of California Santa Barbara Santa Barbara California USA; ^7^ Pacific Biological Station Fisheries and Oceans Canada Nanaimo British Columbia Canada; ^8^ Department of Forest and Conservation Sciences The University of British Columbia British Columbia Vancouver Canada

**Keywords:** climate vulnerability, local adaptation, Pacific salmon, standing variation, structural variants

## Abstract

Understanding the adaptive potential of populations and species is pivotal for minimizing the loss of biodiversity in this era of rapid climate change. Adaptive potential has been estimated in various ways, including based on levels of standing genetic variation, presence of potentially beneficial alleles, and/or the severity of environmental change. Kokanee salmon, the non‐migratory ecotype of sockeye salmon (*Oncorhynchus nerka*), is culturally and economically important and has already been impacted by the effects of climate change. To assess its climate vulnerability moving forward, we integrated analyses of standing genetic variation, genotype‐environment associations, and climate modeling based on sequence and structural genomic variation from 224 whole genomes sampled from 22 lakes in British Columbia and Yukon (Canada). We found that variables for extreme temperatures, particularly warmer temperatures, had the most pervasive signature of selection in the genome and were the strongest predictors of levels of standing variation and of putatively adaptive genomic variation, both sequence and structural. Genomic offset estimates, a measure of climate vulnerability, were significantly correlated with higher increases in extreme warm temperatures, further highlighting the risk of summer heat waves that are predicted to increase in frequency in the future. Levels of standing genetic variation, an important metric for population viability and resilience, were not correlated with genomic offset. Nonetheless, our combined approach highlights the importance of integrating different sources of information and genomic data to formulate more comprehensive and accurate predictions on the vulnerability of populations and species to future climate change.

## 1 INTRODUCTION

In this time of rapid climate change, understanding the adaptive potential of populations and species is pivotal to minimizing the loss of biodiversity. A population is more likely to adapt to rapid changes in the environment if potentially beneficial alleles are already present in the gene pool rather than from new mutations (Barrett & Schluter, 2008). Probabilistically, the chances that an adaptive allele segregates within a population increase with the overall standing genetic variation, a concept that is the foundation of international conservation initiatives aimed at preserving genetic diversity to maximize adaptive potential and minimize extinction risk (Exposito‐Alonso et al., 2022; Kardos et al., 2021; Tsioumani, 2020). However, the relationship between genome‐wide levels of standing genetic variation and adaptive potential is not always clear (Teixeira & Huber, 2021).

As a result, adaptive genetic variation is starting to be more explicitly considered in models aimed at predicting the potential of populations to adapt to climate change (Bay et al., 2018; Capblancq et al., 2020; Layton et al., 2021). Yet, despite great advances in identifying the genomic basis of important ecological traits, an exhaustive understanding of all traits that contribute to fitness remains challenging (Exposito‐Alonso et al., 2022; Kardos et al., 2021). Furthermore, predictive climate models show strong variation in the severity and direction of global climate change (e.g., more severe warming at higher latitudes; IPCC, 2021), resulting in heterogeneous selection pressures and climate vulnerability across the landscape. Thus, an analytical framework that collectively considers genome‐wide and adaptive genetic variation, as well as the environmental factors that affect their distribution across the landscape, could potentially be more informative to accurately estimate the adaptive potential of populations and species than using any one of these parameters alone. This more holistic approach entails: (1) identifying the environmental factors explaining variation in genetic diversity among individuals and populations, which can in turn elucidate the factors constraining population size based on the relationship between nucleotide diversity and effective population size (Tajima, 1983); and (2) using current genotype‐environment associations (GEA) to estimate population “genomic offset” or “genomic vulnerability” (Bay et al., 2018; Fitzpatrick & Keller, 2015), which measure the mismatch between current adaptive genomic variation and the evolutionary change predicted to be necessary to cope with projected climate change.

The study of neutral and adaptive variation has traditionally focused on sequence variation, mostly in the form of single nucleotide polymorphisms (SNPs), while structural variation, including all changes in sequence position, direction, or gains/losses, has been comparatively less studied despite its demonstrated importance (Mérot et al., 2020). In fact, increasing numbers of studies are showing that structural variants (SVs) not only affect a larger proportion of the genome than SNPs (Catanach et al., 2019; Tigano et al., 2020; Tigano & Russello, 2022), but also play an important role in adaptation, both directly as the genetic basis of adaptive traits (Van't Hof et al., 2016) or indirectly as recombination suppressants (Akopyan et al., 2022). As SVs seem to follow different evolutionary trajectories from the rest of the genome, their analysis can add a layer of information on the patterns of diversity and differentiation at the individual and population levels (Mérot et al., 2020). For example, the analysis of copy number variants (CNVs) has revealed different patterns of population structure compared to SNPs as well as strong associations with fitness‐related traits and local adaptations (Cayuela et al., 2021, 2022; Dorant et al., 2020). Nevertheless, structural variation is still largely ignored in studies investigating GEAs and adaptive potential of populations and species in the face of climate change.

Freshwater ecosystems are extremely vulnerable to climate change given the sensitivity of water temperature and flow regimes to atmospheric warming, the limited ability of organisms to disperse to track environmental change, and the synergistic impacts with other stressors, including eutrophication, acidification, and invasive species (Woodward et al., 2010). Freshwater fish are particularly susceptible to these myriad threats, showing the highest extinction rates among vertebrates in the 20th century (Burkhead, 2012). These concerns are compounded by the exploitation of inland fisheries that provide critical food and economic security for individuals and valuable cultural and recreational services to society (Lynch et al., 2016). Given such constraints, genetic change may represent the only biological option for fish species to persist, especially for those unable to migrate or acclimate. Moreover, a broader understanding of adaptive potential can contribute to active management initiatives, such as assisted migration, to help mitigate the impacts of climate change (Chen et al., 2022; Krabbenhoft et al., 2020).

Climate change represents a significant threat to Pacific salmon (*Oncorhynchus* spp.) due to rising water temperatures and changes in river flows (Carlson et al., 2017; Kundzewicz et al., 2008). For example, dramatic decreases in commercial, recreational, and Indigenous subsistence catches over the past 15 years have been attributed to the direct and indirect effects of climate change on salmon survival (Grant et al., 2021). Kokanee are the freshwater resident form of sockeye salmon (*Oncorhynchus nerka*), with populations found across the species' pan‐Pacific distribution. Kokanee are most abundant in western Canada and the United States, from Alaska to the Pacific Northwest, where they have tremendous economic, ecological, and cultural importance (Jacob et al., 2010; Nguyen et al., 2013; Scheuerell et al., 2007). Given that kokanee are landlocked, they may have a more limited capacity to adapt to climate change than their anadromous counterparts (hereafter referred to as “sockeye”) as escaping unfavorable environmental conditions through range shifts (Woodward et al., 2010) or receiving beneficial alleles via gene flow from other populations are options not readily available due to their spatial isolation (Hedrick, 2013; Tigano & Friesen, 2016). In addition to variation in migratory behavior, sockeye and kokanee can be further differentiated into reproductive ecotypes defined by spawning location/substrate, including river/stream spawners, beach/shore spawners, and deep spawners. The three reproductive ecotypes display different local adaptations to their spawning grounds (Moreira & Taylor, 2015; Samad‐Zada et al., 2021; Taylor et al., 2000; Tigano & Russello, 2022; Veale & Russello, 2017a, 2017b) and may also be differentially impacted by climate change. For example, during a heat wave, warming water temperatures may be more buffered in lakes than in streams, and more so in deep waters than in the shallows. Similarly, changes in precipitation would more promptly affect streams than lakes.

Estimating the adaptive potential of kokanee across its range is important to enable evaluation of the relative climate vulnerability of wild populations and to identify populations that are potentially more robust to changing environments for enhancing hatchery production and stocking to support culturally and economically important fisheries. Here, we paired whole genome resequencing data of kokanee sampled from across their Canadian distribution in British Columbia (BC) and Yukon with model‐based and directly measured environmental data to: (1) investigate the environmental factors affecting the distribution of standing genetic variation, including sequence and structural variation; (2) identify the genomic basis of local adaptation based on GEA analyses; and (3) calculate genomic offset for each population to predict climate change vulnerability. Finally, we explore the relationship between genomic offset and levels of standing genetic variation for both sequence and structural variation, and discuss their relative importance for management and climate change adaptation.

## 2 MATERIALS AND METHODS

### 2.1 Sampling and whole genome resequencing

**FIGURE 1:**
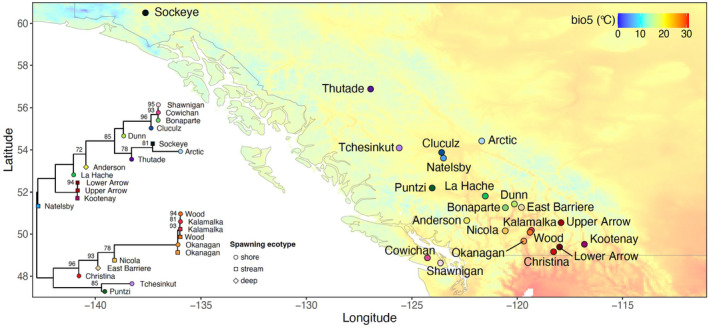
Map of sampled kokanee populations and maximum likelihood phylogenetic tree. Each point in the map represents a lake sampled for this study, and the background represents recent (1970–2000) climate data for bio5, the maximum air temperature of the warmest month. On the left side is the ML tree, constructed based on population allele frequencies, with individuals divided by ecotype in populations where stream‐ and shore‐spawning kokanee co‐occur.

To develop a list of candidate wild populations in BC, we queried the DataBC Catalogue for water bodies with kokanee presence and no stocking history, then interviewed Province of BC fisheries biologists from each fisheries management region for confirmation. In some locations, minimal historic stocking has occurred and is thus expected to have little or no relevance to the genetic integrity of the indigenous kokanee population of interest. This approach resulted in the inclusion of individuals sampled across multiple years, which translates to only 2–3 kokanee generations and is not expected to impact GEA inferences as current climate data are based on 30‐year averages. Specifically, tissue samples were obtained from 264 kokanee individuals from 22 lakes across British Columbia and Yukon spanning 12 degrees of latitude and substantial environmental variation (Figure 1; Table S1); 24 individuals from Okanagan Lake were previously analyzed in Tigano and Russello (2022).

We extracted genomic DNA using a Qiagen DNeasy® Blood and Tissue Kit following the manufacturer's protocol, including an RNAse A treatment. The extracted DNA was quantified using a Qubit 3.0 Fluorometer and the dsDNA High Sensitivity Assay Kit (ThermoFisher Scientific). We generated whole genome resequencing data targeting a minimum average genome coverage of 6×. Library preparation and sequencing were performed at Canada's Michael Smith Genome Science Centre using MGI sequencing technology and 200‐bp paired‐end reads, as detailed in Tigano and Russello (2022).

### 2.2 Variant calling—sequence and structural variation

Before variant calling, we assessed raw data quality and trimmed potential adapter contamination with FASTP (Chen et al., 2018). The trimmed data were mapped to the sockeye reference genome (Christensen et al., 2020) with BWA MEM (Li & Durbin, 2009), and duplicates were removed using SAMBLASTER (Faust & Hall, 2014).

To maximize computational efficiency, we split the genome assembled into chromosomes in 161 10‐Mb sequence blocks and called SNPs in each of these blocks separately using the *mpileup* command in BCFTOOLS (Danecek et al., 2014), filtering SNPs with depth >50 and with mapping and base quality <30. We applied another round of SNP quality filtering using VCFTOOLS (Danecek et al., 2011) and retained only biallelic SNPs (no indels) that had a minimum coverage of three and less than 30% missing data across individuals. We then concatenated the resulting vcf files with *bcftools concat*. As GEA analyses are sensitive to missing data and the effect of linkage disequilibrium, we filtered individuals that had >30% missing data across all filtered SNPs using VCFTOOLS and pruned SNPs that were in high LD (*r* ^2^ > 0.5) within 200‐kb sliding windows using PLINK (Purcell et al., 2007) and VCFTOOLS. Finally, we excluded SNPs that mapped to repetitive areas to further filter paralogous loci.

We called structural variants from the same whole genome resequencing data using an iterative approach implemented in DELLY2 (Rausch et al., 2012). First, we called SVs from each individual's mapped data using *delly call* and merged all SVs called across individuals with *delly merge*. We then genotyped each of these SVs across all individuals jointly with *delly call* and *delly merge* once again. We applied stringent filters for structural variation that included a first round of filtering using *delly filter* and the “germline” setting, then further filtered for SVs that did not obtain the “PASS” flag across individuals using the *bcftools query*. We replaced genotypes that were flagged as “low quality” (i.e., that had lower than three paired‐end reads supporting the variant) as missing data using the *bcftools filter* and filtered SVs with more than 20% missing data across individuals. This missing data threshold, though more stringent than that applied to SNPs, still ensured that we retained all individuals that were included in the final SNP dataset, with the exception of two individuals for which SV calling failed completely.

### 2.3 Environmental data

We downloaded recent (1970–2000) and projected future (2041–2060) data for 19 bioclimatic variables, including air temperature and precipitation variables, from the WorldClim database at a resolution of five arcsmin. We relied on air temperature as a proxy for water temperature, as such data are not available for the water bodies included in our study. Although there can be some disconnect between air and water temperatures due to hydrogeological features such as depth, flow rates, and water sources (e.g., surface or spring‐fed), this approach has some empirical support for salmonids (i.e., studies linking the growth rate of Lake Trout to air temperature; Black et al., 2013; Torvinen et al., 2023) and has been effectively applied in other systems (Andrews et al., 2023; Dallaire et al., 2021). Projected future environmental data were based on the UKESM1‐0‐LL model from the 6th Coupled Model Intercomparison Project (CMIP6) under the two most extreme emission scenarios, RCP2.6 and RCP8.5, which constitute the best‐ and worst‐case scenarios in terms of emissions of greenhouse gases and resulting intensity of global warming, respectively.

We included additional, directly measured variables related to lake geography (elevation, surface area, and maximum depth) and biogeochemistry [surface pH and total dissolved solids (TDS)] that were obtained through the DataBC Catalogue (https://www2.gov.bc.ca/gov/content/data/bc‐data‐catalogue).

### 2.4 Genome‐wide standing genetic variation and differentiation

We estimated sequence and structural standing genetic variation at the individual level as the proportion of heterozygous sites (i.e., the number of heterozygous sites divided by the number of sites actually genotyped in each individual) across SNPs or SVs, respectively, with VCFTOOLS. As sample size varied across populations, we adopted this measure of genetic diversity over nucleotide diversity because it is not affected by sample size and allows comparisons across individuals and populations. To investigate the effect of each environmental factor on individual levels of standing genetic variation, we ran linear mixed effect models where proportion of heterozygous sites was the response variable (fixed effect), each environmental variable was the explanatory variable, and population of origin was the random effect to control for. We tested for significant correlations using ANOVAs and calculated the R‐squared or coefficient of determination for each model using the packages *lmertest* and *MuMIn* in R (Kuznetsova et al., 2017; Oksanen et al., 2022; R Core Team, 2021).

To characterize population structure and compare patterns of differentiation based on sequence and structural variation, we performed an analysis of principal components (PCA) based on the SNPs and SVs filtered datasets. We imputed missing genotypes with the most common genotype across individuals and ran the PCA using the *rda* function in the R package *vegan*. We retained the first three principal components (PCs) and plotted individual PC scores with *ggplot* in R.

To further characterize the phylogenetic relationships among populations, we built a maximum likelihood (ML) tree in IQTREE (Nguyen et al., 2015) using a polymorphism‐aware phylogenetic model (PoMo; Schrempf et al., 2016) based on population site frequency data. For this analysis, we filtered the vcf file to retain only variants with no missing data using VCFTOOLS, converted the resulting dataset into fasta format with the script *vcf2fasta.py* (https://github.com/santiagosnchez/vcf2fasta) coding heterozygous sites using IUPAC ambiguities, calculated population site frequencies with *FastaToCounts.py* (https://github.com/pomo‐dev/PoMo), and ran IQTREE using PoMo, a general time reversible model (GTR) as substitution model, and 1000 ultrafast bootstrap replicates (Hoang et al., 2018). We visualized the consensus tree after midpoint rooting and bootstrapping values with the R package *ggtree* (Yu et al., 2017).

### 2.5 Genotype–environment associations

We used redundancy analysis (RDA), a multivariate approach that analyzes many loci and environmental variables simultaneously, to investigate genotype–environment associations and identify candidate loci underlying local adaptation. RDA has been shown to outperform univariate statistical methods and Random Forest approaches under different evolutionary scenarios and selection pressures and is particularly well suited to identify loci under weak, polygenic selection (Forester et al., 2018).

Before proceeding with the GEA analysis, we performed an environmental variables selection step to remove variables with missing data (e.g., TDS) or those showing strong correlation (*r* ^2^ > 0.7) with other variables (Figure S1), retaining those displaying the highest biological relevance for kokanee. Due to the high correlation among environmental variables (Figure S1), we retained lake surface area, pH, bio5 (maximum temperature of the warmest month), bio6 (minimum temperature of the coldest month), bio15 (precipitation seasonality), and bio16 (precipitation of the wettest quarter); all six variables were standardized and scaled.

To partition genetic variance, we ran a series of partial RDAs (pRDAs), where climate (including the six variables described above), population structure (as summarized by the first three PCs of the population structure PCA, see above), geography (latitude and longitude), and reproductive ecotype (stream/shore and shallow/deep) were either the explanatory or the conditioning variables. We tested all combinations to quantify the proportion of genetic variance explained by each set of variables summarizing climate, population structure, geography, or reproductive ecotype.

To identify loci underpinning local environmental adaptation, we ran a pRDA, including the six environmental variables selected as predictors and individual genotypes as responses. We conditioned this analysis for population structure using the first three PCs of the population structure PCA to minimize spurious GEA, but excluded geography and reproductive ecotypes due to the low proportion of variance explained in the previous step. We identified candidate GEA loci as those with RDA loadings falling on the tail of the distribution on the first three axes with an outlier cut‐off of 3 SD (two‐tailed *p*‐value = 0.0027). We further calculated the correlation coefficients of each candidate locus to each of the six environmental variables and identified the variable showing the strongest associations with each candidate locus. From the set of candidate loci identified (“all candidate loci”), we created a subset of “strong candidate loci” showing strong correlation with a given environmental variable (*r* ^2^ > 0.5). Finally, we assessed the relative importance of the six environmental variables in explaining genetic variation across all variants, only candidate loci, and only strong candidate loci with the *ordiR2step* function in *vegan* (Oksanen et al., 2022).

We functionally annotated strong sequence and structural variant outliers by extracting either the genes on which the variants fell or the closest gene to the variants that were positioned in an intergenic area using BEDTOOLS functions (Quinlan & Hall, 2010) implemented in the R package *valr* (Riemondy et al., 2017). To better understand the nature of phenotypic environmental adaptation, we performed a Gene Ontology (GO) enrichment analysis of the strong sequence outliers only with the online app ShinyGO 0.76.3 (Ge et al., 2020) using zebrafish as the reference species, a FDR cut‐off of 0.05, and three different databases, including: GO Biological Process (larger processes accomplished by multiple molecular activities); KEGG pathways (metabolic pathways most affected by adaptive genomic differentiation); and Phenotype ZFIN (phenotype data associated with zebrafish genes).

### 2.6 Adaptive landscape

To characterize the “adaptively enriched genetic space,” we ran pRDAs using the same model as above, including the six environmental variables as explanatory variables and population structure as a conditioning factor, but with only subsets of candidate loci (“strong sequence outliers” or “all structural outliers”) as response variables. As surface area and pH are idiosyncratic features of each body of water, with the former not varying over short evolutionary scales and the latter lacking data on projected change for our study system (despite an expected overall acidification of freshwater bodies; Schindler, 1997), we excluded these variables and the loci most strongly associated with them for downstream analyses. We ran another adaptively enriched pRDA using only the four remaining variables [two temperature variables (bio5, bio6) and two precipitation variables (bio15, bio16)] and the loci associated with these variables. We then calculated the adaptive index across the landscape, a measure of adaptive genetic similarity as a function of the environmental variation across space, based on the scores of the four temperature and precipitation variables on the first two RDA axes (Steane et al., 2014).

We estimated the genomic offset for each sampled population using a gradient forest (GF) approach as implemented in the R package *gradientforest* (Breiman, 2001). This analysis is based on a machine‐learning regression tree approach that maps patterns of genetic variation using nonlinear functions of environmental gradients. First, we assessed the relative importance of all environmental variables, including all those excluded in the RDA due to strong correlations, and calculated *r* ^2^ weighted importance values for each variable using GF models with 500 trees and a correlation threshold of 0.5, all 28 environmental variables as predictors, and either the strong sequence outliers or all structural outliers as response variables. Bio5 was the variable with the highest and second highest *r* ^2^ weighted importance in explaining patterns of sequence and structural adaptive genetic variation, respectively, and was highly correlated (*r* ^2^ = 0.95) with bio10, showing similar weighted importance in the sequence variation analysis. Consequently, we calculated genomic offset based on the change in bio5 predicted for the period 2041–2060 under the best‐ and worst‐case climate change scenarios (RCP2.6 and RCP8.5). First, we transformed bio5 from each of the three datasets (recent, future best, and future worst) into genetic importance using the turnover function (Breiman, 2001). Then, for each grid cell, we calculated genomic offset as the Euclidean distance between recent and future genetic importance values (Ellis et al., 2012) and extracted genomic offset values for each population.

To test whether reproductive ecotype contributes to the climate change vulnerability of a population, we recalculated allele frequencies at the strong sequence outlier loci for each ecotype separately in the locations where both ecotypes co‐occur (Okanagan, Wood, and Kalamalka) and repeated the genomic offset analysis as described above.

## 3 RESULTS

### 3.1 Genome‐wide standing genetic variation and differentiation

Genomic coverage of mapped reads after quality filtering was 6.3× with a mapping rate of 94.1% on average (average data per sample = 14.9 Gbases). We identified a total of 975,527 SNPs and 9422 SVs across 224 individuals based on our stringent filtering criteria and found substantial variation in levels of genetic diversity across individuals: sequence heterozygosity (% of heterozygous sites) varied by 16% (35%–51%) at the individual level and by 9% at the population level (37%–46%), while structural heterozygosity varied by 24% (25%–48%) at the individual level and by 12% (27%–39%) at the population level. Sequence variation, measured as the proportion of individual heterozygous sites, was significantly correlated with nine out of the 28 environmental variables tested (*p* < 0.05; Table 1), with bio6, the minimum temperature of the coldest month, showing the strongest correlation (*p* = 0.006 and *r* ^2^ = 0.19). All environmental variables showing significant associations were related to temperature; precipitation did not appear to be a significant predictor of levels of standing sequence genetic variation in kokanee (Table 1). Structural genetic variation showed significant associations with 12 out of the 28 variables tested, with notable differences compared to sequence variation including: (1) the two variables showing the strongest associations were bio10 and bio5, mean temperature of warmest quarter and maximum temperature of warmest month (*p* = 0.0023 and 0.0026, *r* ^2^ = 0.33 and 0.32, respectively); and (2) lake size variables (surface area, depth, and volume) showed significant correlations with structural variation but not with sequence variation (Table 1). Precipitation was not a significant predictor of structural variation, similar to what was found for sequence variation (Table 1).

**TABLE 1:** Results of linear mixed effect models to investigate the relationship between genomic heterozygosity (sequence and structural) and environmental variation.

	Sequence variation		Structural variation	
	*R* ^2^	*p*‐value	*R* ^2^	*p*‐value
Longitude	0.0399126	0.253	**0.1521631**	**0.03531**
Latitude	**0.121587**	**0.0366**	**0.1608291**	**0.02752**
Elevation	**0.1101613**	**0.0363**	**0.1410887**	**0.02763**
Surface area	0.001822102	0.8282	**0.168237**	**0.04369**
Degree days	**0.154019**	**0.02049**	**0.2960658**	**0.002088**
TDS	0.01774042	0.4798	0.005463571	0.7248
pH	0.006860478	0.6603	7.310497e−05	0.9669
Depth	0.0795660	0.1103	**0.2227124**	**0.01058**
Annual mean temperature (bio1)	**0.1537017**	**0.0221**	**0.1938168**	**0.01847**
Mean diurnal range (bio2)	**0.1616045**	**0.0105**	0.06624291	0.1482
Isothermality (bio3)	0.002647736	0.7601	0.0251414	0.3832
Temperature seasonality (bio4)	**0.1203755**	**0.02702**	0.01593077	0.4726
Max temperature of the warmest month (bio5)	0.067112	0.1724	**0.3224192**	**0.0026**
Min temperature of the coldest month (bio6)	**0.189883**	**0.006467**	**0.1569349**	**0.0271**
Temperature annual range (bio7)	**0.1411615**	**0.01544**	0.02899479	0.3298
Mean temperature of the wettest quarter (bio8)	0.0003701677	0.9187	0.002207944	0.8191
Mean temperature of the driest quarter (bio9)	0.03640297	0.2424	0.05603867	0.181
Mean temperature of the warmest quarter (bio10)	0.1211831	0.05915	**0.3252551**	**0.002336**
Mean temperature of the coldest quarter (bio11)	**0.1673324**	**0.01156**	0.1282612	0.04826
Annual precipitation (bio12)	0.06328889	0.105	3.858704e−06	0.9905
Precipitation of the wettest month (bio13)	0.07481418	0.07382	0.0008639013	0.8578
Precipitation of the driest month (bio14)	0.03706275	0.2537	0.04194532	0.2659
Precipitation seasonality (bio15)	0.04017974	0.2277	0.001093043	0.8547
Precipitation of the wettest quarter (bio16)	0.0696947	0.08611	0.000244928	0.9243
Precipitation of the driest quarter (bio17)	0.0173979	0.4527	0.008421016	0.6331
Precipitation of the warmest quarter (bio18)	0.03547063	0.3089	0.1100306	0.09444
Precipitation of the coldest quarter (bio19)	0.07656698	0.06917	0.00141505	0.8179
Volume	0.007473841	0.6633	**0.1752996**	**0.04114**

*Note*: Bolded values are significant (*p* < 0.05).

**FIGURE 2:**
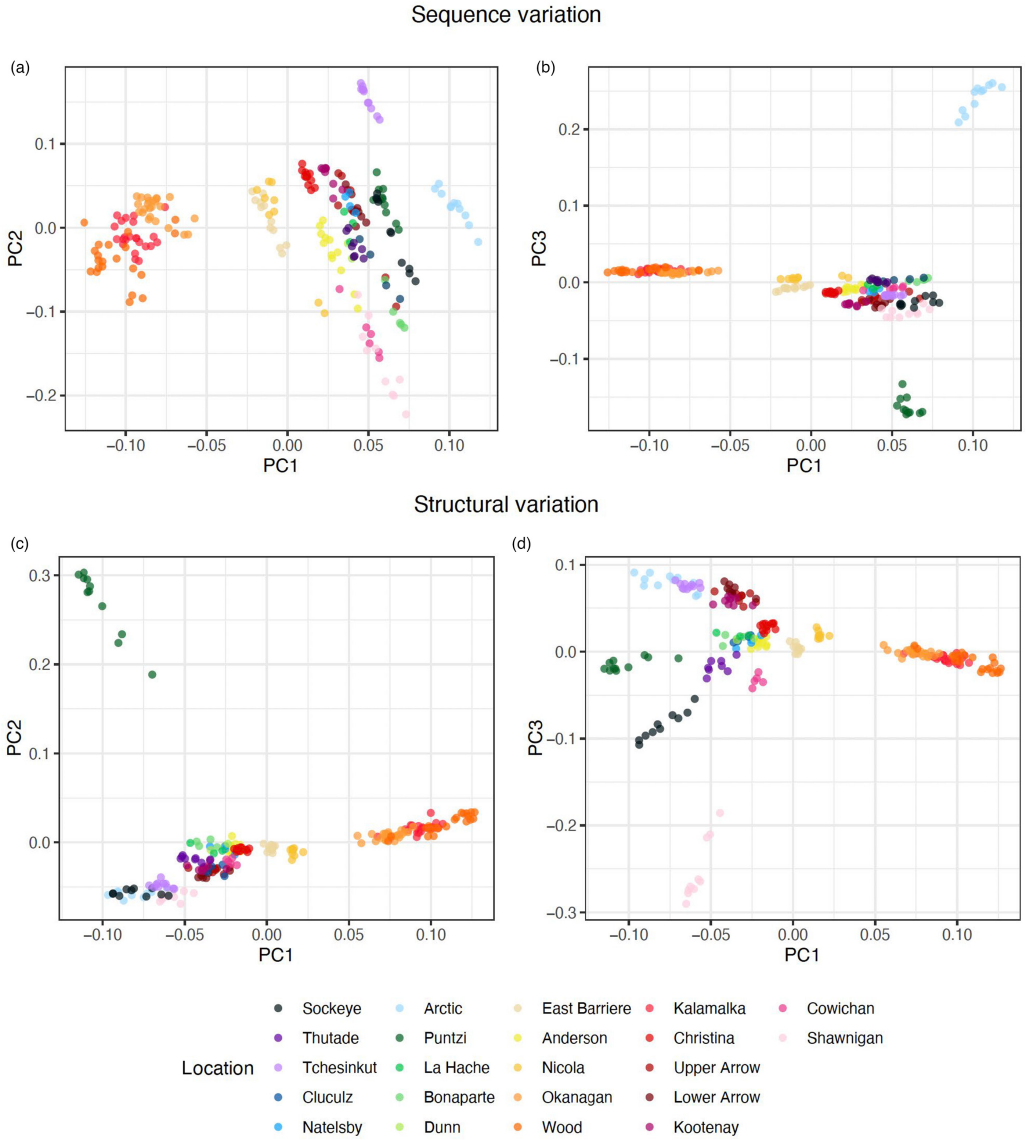
Principal component analysis (PCA) plots showing population differentiation based on sequence (a, b) and structural (c, d) variation along PCA axes 1 and 2 (a, c) and axes 1 and 3 (b, d).

The PCAs of sequence and structural variation both showed individuals clustering mostly by sampling location, with kokanee from adjacent lakes in the Okanagan Basin (Okanagan, Wood, and Kalamalka) forming a group markedly distinct from the rest of the samples (Figure 2). However, populations did not form distinct groups based on river basin or geographical proximity in the sequence PCA, with Arctic, Tchesinkut, and Puntzi constituting the most distinct populations from the rest of the samples that were relatively widespread across PC1 and PC2 (Figure 2a,b). In contrast, samples formed three distinct groups in the structural PCA, with two exceptions: (1) Puntzi that was isolated from the rest on PC2 (Figure 2c); and (2) Shawnigan that was most distinct on PC3 (Figure 2d).

Phylogenetic relationships based on the ML tree were mostly concordant with grouping based on the sequence PCA (Figures 1 and 2a,b). Okanagan Basin lakes formed a distinct clade most closely related to the southern interior BC lakes, with the exception of Upper and Lower Arrow and Kootenay in the Kootenay Mountains region, which formed a distinct clade more closely related to kokanee from more northern locations. However, Puntzi and Tchesinkut clustered with the southern interior BC clade rather than with the surrounding populations, such as Cluculz, Natelsby, or La Hache. Shawnigan and Cowichan, both on Vancouver Island, clustered together and were closely related to Bonaparte and, to a lesser extent, Cluculz. Arctic and Tchesinkut, which were most isolated from the rest in the PCA space, showed the longest branches in the ML tree (Figure 1). Overall, with a few exceptions, geographic proximity did not seem to be a good predictor of genetic similarity in the ML tree.

### 3.2 Genotype‐environment associations

The variance partition analysis from the full pRDA model showed that climate, population structure, geography, and ecotype explained 18% of the sequence genomic variance and 25% of the structural genomic variance. The climate pRDA model explained more variance (6% and 7% of sequence and structural variation, respectively) than any other variance partitioning model (Table S2). The RDA model including only climate showed the same hierarchy of relative importance of the six environmental variables across the sequence and structural genomic datasets, with bio5 (the maximum temperature of the warmest month) consistently explaining the highest genomic variance. Given the strong population structure shown in the PCA that explained 4%–5% of the genomic variance, we based the detection of outlier loci on a model that included the six environmental variables as explanatory variables, conditioned for population structure. This model explained 7% and 9% of the sequence and structural genomic variance, respectively.

**FIGURE 3:**
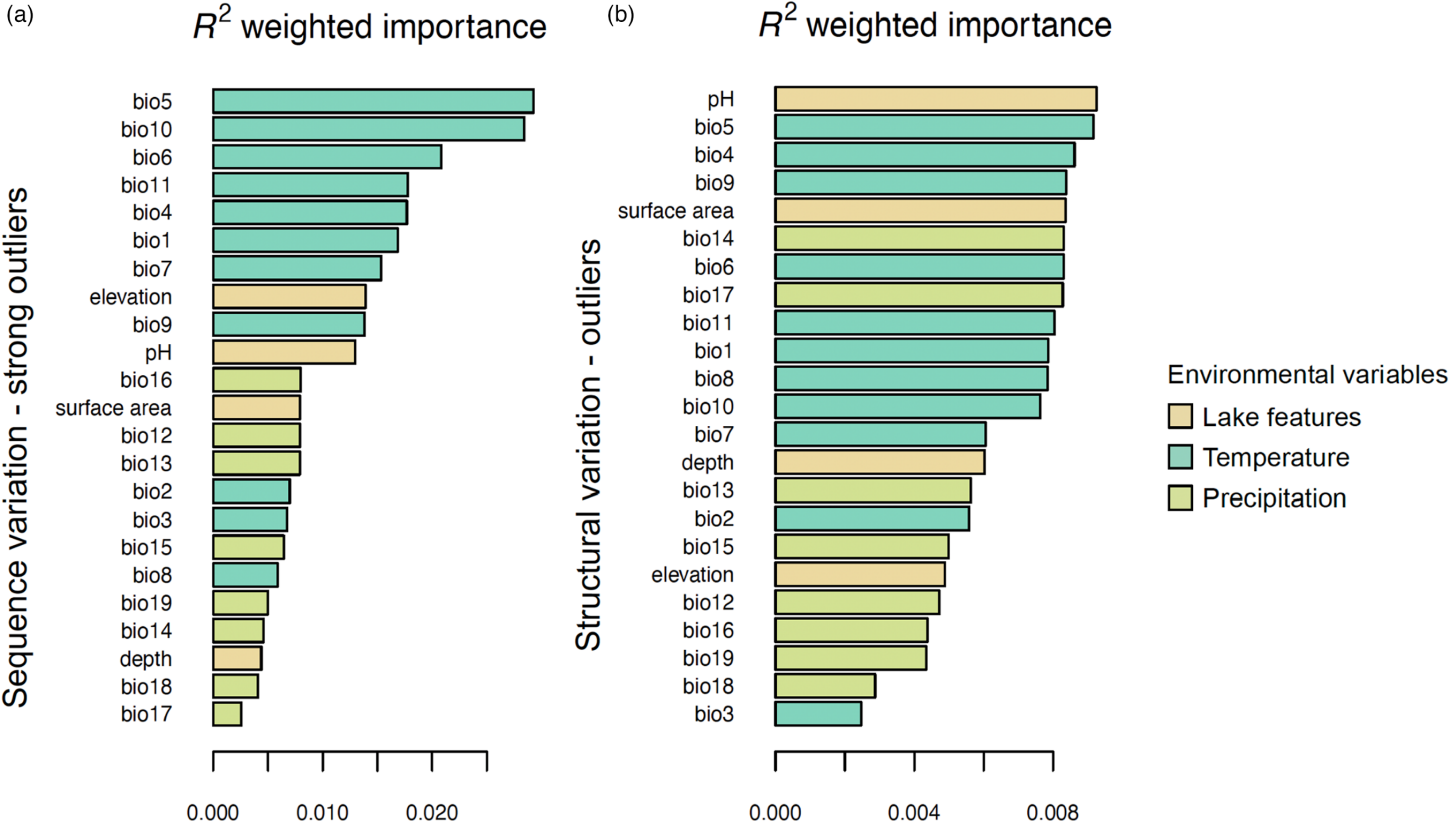
Histograms of the *R* ^2^ weighted importance of environmental variables in explaining adaptive variation. Strong sequence outliers only on the left (a) and all structural outliers on the right (b).

Based on this latter model, we identified a total of 57,468 outlier SNPs and 329 outlier SVs as candidate variants for local adaptation. Bio6 (minimum temperature of the coldest month) was the variable showing the strongest association with the highest number of outlier SNPs (*n* = 14,624), followed by pH (*n* = 14,197). This pattern was flipped for structural variation, with pH being the variable showing the strongest association with the highest number of outlier SVs (*n* = 94), followed by bio6 (*n* = 76). Strong sequence outliers (*n* = 1444) showed a sharp difference in their distribution across environmental variables compared to all sequence outliers, with bio5 being the variable with the highest number of most strongly associated outliers (*n* = 521, 36% of strong sequence outliers). Bio16 (precipitation of the wettest quarter) and surface area had the lowest number of most strongly associated sequence outliers, whether all outliers or only strong outliers were considered. Only five outlier SVs, including three insertions, one duplication, and one deletion, showed correlations stronger than 0.5 (“strong SV outliers”), two of which were associated with bio5 and three with bio6. The GF analysis based on strong sequence outliers was consistent with results from the pRDAs, revealing extreme hot temperatures (bio5 and bio10), followed by extreme cold temperatures (bio6 and bio11), as the most important variables explaining adaptive sequence variation in kokanee and that precipitation is generally not an important predictor (Figure 3). In contrast, the hierarchy and distribution of the importance of environmental variables in explaining adaptive structural variation were quite different; pH was the most important variable (followed by bio5), and the *r* ^2^ of the most important variables was one order of magnitude lower than the most important variables for adaptive sequence variation (Figure 3).

We identified a total of 1234 genes associated with strong sequence outliers, though we were not able to retrieve the function for 106 of these genes. The GO enrichment analysis showed that these genes were significantly enriched for five biological processes, four KEGG pathways, and 53 phenotypes (Table S3). The function that was consistently enriched across databases was related to the development of the nervous system.

We identified four coding genes associated with strong SV outliers: *dok7* was most strongly associated with bio5 and is involved in the development of neuromuscular junctions, while *plb1*, *rpgra*, and *lefty2*, involved in lipid absorption, retinal development and cell death, and left–right asymmetry determination of organ systems during development, respectively, were most strongly associated with bio6. Two of these genes, *dok7* and *plb1*, were also associated with strong SNP outliers.

### 3.3 Adaptive index

**FIGURE 4:**
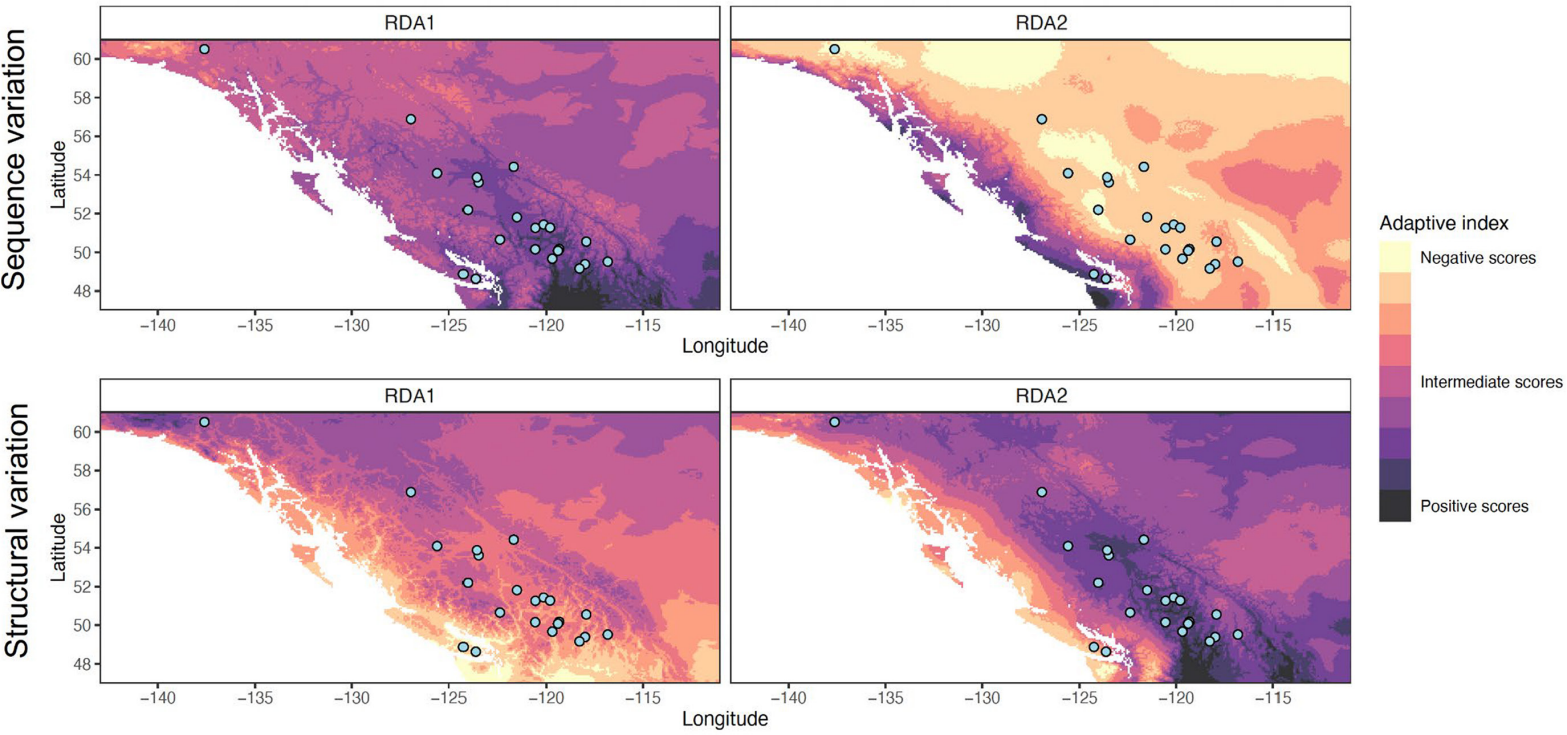
Adaptive index plots calculated from sequence variation (top) and structural variation (bottom) based on RDA axis 1 (left), most strongly associated with temperature variables, and axis 2 (right), most strongly associated with precipitation variables. Circles represent sampled locations.

The first two axes of the adaptively enriched RDAs, which were based on models that included outlier variants and the four climate variables only, explained most of the adaptive genetic variance in both the sequence and structural variant datasets (37% and 25% in the sequence RDA and 47% and 24% in the structural RDA). In both RDAs, RDA axis 1 (RDA1) was most strongly associated with the two temperature variables (bio5 and bio6), while RDA axis 2 (RDA2) was most strongly associated with the two precipitation variables (bio15 and bio16), which was reflected in the calculation of the adaptive index along the two RDA axes (Figure 4). Specifically, RDA1 differentiated sampled populations in BC and Yukon on the basis of their thermal regimes, with extreme values in the southern and northwestern parts of the range, while RDA2 summarized the strong precipitation cline from the Pacific coast to interior BC. These results were consistent across both sequence and structural variation, albeit with opposite extremes in adaptive index score (i.e., negative values in colder and drier areas in the sequence RDA and warmer and wetter areas in the structural RDA; Figure 4).

### 3.4 Genomic offset

**FIGURE 5:**
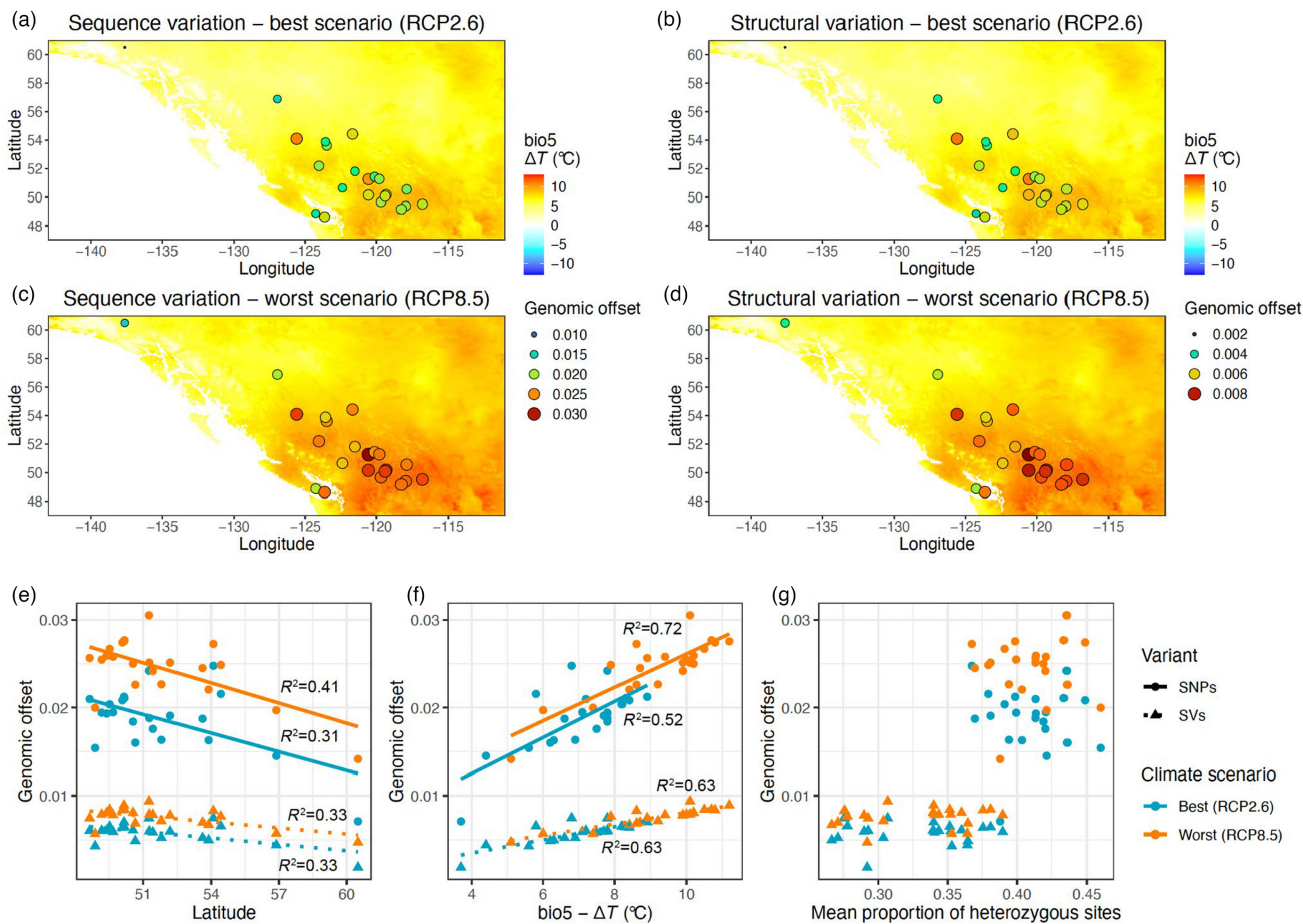
Top, genomic offset estimates based on sequence (a, c) and structural variation (b, d) according to the best‐case (RCP2.6; a, b) and worst‐case climate change scenarios (RCP8.5; c, d). The map background shows changes in the maximum temperature of the warmest month (bio5 Δ*T*) predicted for the period 2041–2060. Below, plots showing the correlation, or lack thereof, of genomic offset estimates with latitude (e), bio5 Δ*T* (f), and genomic heterozygosity (g).

Maps showing the predicted change in maximum temperature of warmest month (bio5 Δ*T*) revealed an uneven degree of change across the kokanee range, with the highest increase in southern Interior BC in the area bounded by the Kootenay/Columbia Mountains in the east, Interior Mountains in the north, and the Coast Mountains in the west (Figure 5).

The genomic offset estimates based on sequence and structural variation were highly concordant (*r* ^2^ = 0.95 and 0.94 for the best‐ and worst‐case climate change scenarios, respectively; Figure 5). Sockeye and Thutade, the two northernmost locations, and Cowichan, on Vancouver Island, had the lowest genomic offset values across climate change scenarios and genomic data types (Figure 5; Table S4), while Bonaparte and Nicola consistently exhibited some of the highest genomic offset values across analyses (Figure 5; Table S4). Tchesinkut and Arctic appeared to be most vulnerable to increases in extreme warm temperatures in the best‐case climate change scenario, and Kalamalka in the worst‐case climate change scenario (Figure 5; Table S4). Sockeye, Thutade, and Cowichan are expected to experience the lowest increase in bio5 and Nicola the highest, according to both climate change scenarios. Overall, increase in bio5 was a strong predictor of genomic offset estimates (*r* ^2^ = 0.52 and 0.72 for sequence variation under the best‐ and worst‐case climate change scenarios, respectively; *r* ^2^ = 0.63 for structural variation, under both the best‐ and worst‐case climate change scenarios; Figure 5f), more so than latitude (*r* ^2^ = 0.31 and 0.41 for sequence variation under the best‐ and worst‐case climate change scenarios, respectively; *r* ^2^ = 0.33 for structural variation, under both the best‐ and worst‐case climate change scenarios; Figure 5e). Genomic offset estimates based on any model were not correlated with levels of standing genetic variation, sequence, or structural, respectively (*p* > 0.05; Figure 5g).

At a finer level, we found no relationship between reproductive ecotype and climate change vulnerability in lakes where they naturally co‐occur within Interior BC. Stream and shore spawners had the same genomic offset in Okanagan, shore spawners had a higher genomic offset in Kalamalka, and stream spawners had a higher genomic offset in Wood, indicating that spawning location may not generally predict vulnerability to climate change, at least within this region, based on either sequence or structural variation.

## 4 DISCUSSION

Our work addresses fundamental questions about the relative importance of genome‐wide levels of standing variation and adaptive variation, both sequence and structural, to predict the evolutionary potential of populations to adapt to climate change and the environmental factors that exert the strongest selection in kokanee, a freshwater salmonid with tremendous cultural and economic importance. We found that extreme warm temperature was the most important environmental factor among those examined for explaining levels of standing genetic variation, acting as a selective agent for local adaptation, and predicting genomic offset in kokanee. As climate vulnerability inferred from levels of standing genetic variation and adaptive genetic variation were not correlated, below we discuss the relative importance of each and how they both provide important information for active management and climate change mitigation.

### 4.1 Complex population structure

Population structure can be a strong confounding factor in GEA analyses, especially when genetic differentiation, geographic distance, and environmental distance covary (Rellstab et al., 2015). Our analyses show that population structure in kokanee from BC and Yukon is complex, but it is not clearly associated with geographic or environmental distance. For example, kokanee largely grouped by lake, with a weaker signature of geographic structure at a broader, regional level (e.g., Okanagan Basin lakes). Yet, geographic proximity did not appear to be a good predictor of genetic similarity overall, probably due to lake colonization history following the Last Glacial Maximum (Taylor et al., 1996). For example, Christina Lake, geographically between the Okanagan Basin (Okanagan, Wood, and Kalamalka) and the Kootenay region (Arrow and Kootenay), grouped more closely with lakes further west (i.e., Nicola and East Barriere) in the ML tree, while Cluculz was more closely related to Bonaparte (~350 km away) and the Vancouver Island populations (~500 km away) than to surrounding populations. Likewise, there were no signatures of a broader structure based on ecotype, as previously shown (Frazer & Russello, 2013; Lemay & Russello, 2012; Moreira & Taylor, 2015; Samad‐Zada et al., 2021; Veale & Russello, 2017b). Our GEA analyses controlled for this marked and complex population structure to avoid spurious genotype–environment association. At the same time, the uncoupling of genetic differentiation and geographic/environmental distance among sampling locations strengthens the robustness of our GEA results.

### 4.2 Temperature is a strong driver of levels and distribution of genomic variation

Temperature was consistently highlighted across analyses as the strongest environmental factor explaining the distribution of genomic diversity and adaptive genomic variation. Specifically, we found that both sequence and structural diversity increased with temperature, with the minimum temperature of the coldest month (bio6) and the mean temperature of the warmest quarter (bio10) being the strongest predictors, respectively. These results suggest that temperature is an important limiting factor of population growth in kokanee, as has been found in other systems (Savage et al., 2004). In sockeye, the deleterious effects of increasing water temperatures are well documented (Eliason et al., 2011; Whitney et al., 2013). Low temperatures and temperature variance can also affect fitness and limit population growth. For example, an increase in winter temperatures increased fitness and population growth in a migratory songbird (*Passerculus sandwichensis*; Woodworth et al., 2017), while the American lobster (*Homarus americanus*) showed strong genomic signatures of selection in response to sea surface temperature variance (Dorant et al., 2020). Although temperature explained a large proportion of the characterized standing genetic variation, factors related to the evolutionary history of kokanee, including colonization history and hybridization with sockeye, could potentially contribute to the observed variation in diversity; the role of such factors remains to be tested.

We had expected stronger relationships between genomic variation and precipitation given the latter's influence on stream discharge, yet no such associations were found. In migratory sockeye, increases in stream discharge can cause higher energy expenditure (Hinch & Rand, 1998), resulting in decreased survival (Rand et al., 2006), while low streamflow can cause low oxygen conditions and pre‐spawning mortality (Tillotson & Quinn, 2017). In kokanee, air temperature may have a stronger influence on hydrology than precipitation during key times in the kokanee life cycle (Pitman et al., 2020).

Our GEA analyses also showed that environmental variation (climate + lake features) had a greater effect on the distribution of genomic variation in kokanee than population structure, geography, or ecotype, and that the maximum temperature of the warmest month (bio5) was the variable explaining the highest proportion of genomic variance in all models, including those based on sequence or structural variation (all variants or only outliers). Given that environmental variables were thinned prior to the RDA to minimize correlation, it is possible that a removed variable may have exerted a more significant influence on observed patterns of variation. However, bio5 and, to a lesser extent, bio6 were consistently highlighted as the most important variables across all analyses, including those that included all environmental variables without a thinning step (e.g., GF). As such, these findings indicate that extreme hot temperatures are likely a strong source of selection in kokanee. Migratory sockeye show physiological adaptation in thermal tolerance, with differences among populations associated with migration conditions such as distance, elevation gain, and river temperature and flow (Eliason et al., 2011). In the absence of specific physiological data regarding thermal tolerance, the widespread selective signature of temperature on both genomic diversity and adaptive variation in kokanee genomes strongly suggest that the resident form of *O. nerka* may also be adapted to local thermal regimes, in particular extreme warm temperatures.

We expected polygenic signatures of selection in kokanee, given that environmental variation can be a multifaceted source of selection. In salmon, temperature alone can affect survival, phenology (Crozier et al., 2011), growth rates (Martins et al., 2012), prevalence of diseases (Wagner et al., 2005), and oxygen content (Pörtner & Farrell, 2008). Our analyses based on RDA enabled us to detect expected polygenic signatures of selection and to gain insights into the genetic architecture of environmental local adaptation based on the number of outliers associated with each variable and the strength of their correlation. For example, here, bio6 and pH were associated with the highest number of sequence and structural variants when all outliers were considered, but bio5 was associated with 36% and 40% of the strong sequence and structural outliers, respectively. These findings suggest that while bio6 and pH had a more widespread signature of selection in the genome, likely due to more loci of weak effect, selection exerted by bio5 may be stronger and/or involve fewer loci of larger effect.

The polygenic nature of environmental adaptation in kokanee was further reflected in the variety of biological processes, metabolic pathways, and phenotypes significantly enriched. We identified many terms related to body structure as well as organ and nervous system development. Significant enrichment of terms associated with anatomical structure morphogenesis and body size/shape suggests that observed body size variation (Taylor et al., 1997) may occur throughout the range of kokanee as a response to environmental variation. Other enriched phenotypes related to blood circulation, heart size, and pericardium were consistent with adaptation to the local thermal environment reported at the cardiorespiratory level in sockeye (Eliason et al., 2011). Enriched phenotypes related to the eye, brain, and development of the nervous system have been attributed to differences in visual habitat between streams and lakes when comparing stream‐ and shore‐spawning kokanee (Tigano & Russello, 2022). As stream‐spawning kokanee in our dataset primarily occur in the Okanagan Basin (Okanagan, Wood, and Kalamalka) and Kootenay region (Arrow and Kootenay), all among our southernmost sampled locations, our GEA analyses may have either captured ecotypic differentiation or indicate that colder and/or wetter environments tend to favor shore spawning over stream spawning, or both. An in‐depth characterization of the evolutionary history of kokanee is needed to better understand the interaction between local adaptations associated with reproductive behavior and environmental variation.

### 4.3 Climate vulnerability in kokanee varies throughout its range

Identifying extreme hot temperatures as the climate variable with the strongest signal of selection indicates that increasing summer temperatures and heat waves may be the biggest threats posed by climate change to kokanee. Climate models for the next 20–40 years predict that kokanee populations included in this study will experience an increase in extreme air temperatures between 3.7–8.9 and 5.1–11.2°C according to the best‐ and worst‐case scenarios, respectively. It is important to note, however, that water warming is harder to predict due to the idiosyncratic hydrogeological features of different bodies of water. With this in mind, our genomic offset estimates were highly correlated with expected increases in extreme air temperatures and indicated that populations in the northern range, such as Sockeye and Thutade, are less vulnerable to climate change than populations in southern BC, such as Bonaparte and lakes in the Okanagan Basin and Kootenay region, where temperatures will increase faster. From a physiological standpoint, the thermal optimum of these northern populations may be farther from their thermal tolerance upper limit than more southern populations, which are already living in much warmer waters, making them more resistant to increases in water temperature. Furthermore, if low temperature limits population growth, as suggested by the positive correlation between levels of genomic diversity and temperature (bio5 and/or bio6, depending on the type of genomic variation), northern populations of kokanee may even benefit from an increase in water temperature due to reduced ice cover time, higher lake productivity, and a longer period of optimal thermal range of temperatures for growth. However, these hypotheses remain to be tested with physiological assessment of thermal tolerance and resilience; in fact, populations of cutthroat trout (*O. clarkii*) show differences in thermal tolerance depending on the maximum temperature they experience (Anlauf‐Dunn et al., 2022). These results together suggest that the range of kokanee may contract northward in the future, as projected for other salmonids (Abdul‐Aziz et al., 2011).

Ecotype was not a good predictor of climate vulnerability in kokanee at both broad and local scales. Stream spawners did not appear to be more vulnerable to climate change than shore spawners, based on our genomic offset estimates for populations sampled throughout BC and Yukon. Moreover, there was no clear pattern that emerged when comparing genomic offset estimates in the three locations where stream and shore spawners naturally co‐occur in the Okanagan Basin. Yet, available climate data are limited to air temperature at a coarse geographic scale that also does not account for microclimate variability, including differences between in‐lake and riverine habitats. The degree to which any disparity may differentially influence climate vulnerability among reproductive ecotypes requires further study. Direct measures of environmental variation in freshwater systems would be particularly important for obtaining more accurate climate vulnerability predictions in general and specifically for the different reproductive and migratory ecotypes.

The predictive power of genomic offset estimates on fitness effects is increasingly being assessed through experimental and simulation studies, showing promising results (Láruson et al., 2022; Lind et al., 2023). It is important to note, however, that genomic offset estimates are still only relative measures of climate vulnerability and do not provide information on the absolute fitness effects of climate change. Nevertheless, experimental studies in sockeye have demonstrated the deleterious fitness effects of increases in temperature on embryo survival (Whitney et al., 2013), swimming capability and aerobic scope (Eliason et al., 2011), and additional stressors such as catch‐and‐release events (Gale et al., 2011). Moreover, recent evidence of severe lesions and fungus infections in sockeye caused by extreme water temperature in the Columbia River (Columbia Riverkeeper, unpublished) following a 2021 heat dome event in western North America further suggests that the kokanee populations showing high relative genomic offset will be heavily affected by rising water temperature.

### 4.4 Standing genetic variation versus adaptive variation for climate change adaptation

The relationship between genomic offset estimates, standing genetic variation, and climate vulnerability also requires further investigation. For example, genomic offset estimates in another salmonid, Arctic charr (*Salvelinus alpinus*), were negatively correlated with latitude and nucleotide diversity, so that southern populations with the highest genomic offset estimates and the lowest genetic diversity were unequivocally deemed the most vulnerable to rising temperatures (Layton et al., 2021). In kokanee, we similarly found that latitude was a significant predictor of genomic offset, though weaker than bio5 Δ*T*; however, no such relationship was found for genomic diversity. Predictions of adaptive potential based on levels of genomic diversity or genomic offset estimates are therefore discordant in kokanee. Given the limitations of both metrics to identify the most vulnerable populations, an approach that considers both may help in obtaining a more thorough assessment (Rellstab et al., 2021). Yet reconciling the contrasting signals from the two measures appears less straightforward. For example, the relative importance of adaptive versus overall genomic variation may vary spatially and temporally and depend on levels of connectivity among populations.

For isolated populations that cannot rely on the input of beneficial alleles from other populations, such as kokanee, the first line of defense against increasing selection pressures due to climate change may be adaptive variation. In these cases, genomic offset estimates based on both adaptive variation and the degree/nature of change may, in fact, be a good measure of climate vulnerability. However, vulnerable populations may experience higher mortality and/or lower fitness, which in turn may initiate a positive feedback loop whereby decreases in population sizes may eventually lead to reductions in standing genomic variation and the resulting consequences for long‐term persistence, including fixation of deleterious alleles, inbreeding depression, and overall reductions in adaptive genomic variation. Additionally, it is important to consider that climate change is only one of many threats to freshwater ecosystems (Reid et al., 2019). Pollution, habitat degradation, diseases, acidification, and the introduction of non‐native species may compound the deleterious effects of climate change in ways that are hard to predict and incorporate into predictive models and genomic offset calculations. Given this uncertainty, maintaining healthy levels of overall standing genetic variation should remain a priority, not only to avoid the consequences of genetic variation loss but also to maximize the presence of potentially advantageous alleles in a changing environment in the gene pool.

### 4.5 Sequence versus structural variation

Climate vulnerability estimates based on sequence and structural variation were highly concordant, and two of the four genes associated with strong SV outliers were also associated with SNP outliers. In contrast, patterns of population structure, levels and drivers of standing genetic variation, and the relative importance of environmental factors associated with adaptive variation differed markedly between analyses based on either sequence or structural variation. For example, although the relative placement of southern BC populations was generally concordant in the sequence and structural PCA, grouping of populations from Vancouver Island and more northern locations differed based on the type of genomic variation examined. Likewise, lake size and pH were significant predictors of overall and adaptive structural variation, respectively, while having markedly limited associations with any form of sequence variation.

The different results from the analysis of sequence and structural variation could be due to different evolutionary rates between different types of mutations. Although the evolutionary rate of SVs is still poorly understood (Mérot et al., 2020), the rate of point mutations in humans is generally higher (~30 mutations per generation; Roach et al., 2010) than for structural variants (0.3 SVs per generation; Collins et al., 2020). However, SVs generally affect a larger proportion of the genome than SNPs in many species (Catanach et al., 2019; Feulner et al., 2013; Tigano et al., 2020), including kokanee (Tigano & Russello, 2022), likely due to their size affecting more sequence than point mutations per generation. Moreover, sequence and structural variation are governed by different underlying molecular mechanisms. In our dataset, structural variation showed higher variation in heterozygosity than sequence variation, both at the individual and population levels. Furthermore, the correlation between levels of sequence and structural standing variation was moderate (*r* ^2^ = 0.49), suggesting that their evolution is at least partially decoupled. Taken together, these observations may help explain the different patterns of population structure and how different types of genetic variants can exhibit different signatures of environmental selection. In the American lobster, for example, population structure and significant associations with sea surface temperature variance inferred from CNVs but not SNPs (Dorant et al., 2020) were attributed to their faster evolutionary dynamics, which have been implicated in abrupt chromosomal reshuffling (Thybert et al., 2018) and rapid adaptation (Reid et al., 2016) in other species. Though characterizing evolutionary dynamics and rates of structural variation remains an active area of inquiry (e.g., Bertolotti et al., 2020; Collins et al., 2020; Ruggieri et al., 2022), further research is needed to better understand the commonalities and idiosyncrasies between sequence and structural variation and their relative roles in adaptation and speciation (Mérot et al., 2020).

## 5 CONCLUSIONS

Assessing the vulnerability of populations and species to climate change is pivotal for management and conservation. This information can be used to inform the creation of protected areas, guide habitat restoration, prioritize research and monitoring resources, and design assisted migration strategies (Chen et al., 2022; Jeffery et al., 2022). In kokanee, stocking for recreational fishing is a common practice in many parts of its range and represents a potentially viable tool to mitigate the effects of climate change on vulnerable populations. The identification of appropriate source populations is central to such active management strategies to target appropriate broodstock and prevent introductions of maladapted individuals that could further reduce the fitness of local populations in the short‐term and long‐term (Chen et al., 2022). Experimental manipulations to assess population‐specific physiological responses to environmental change (e.g., increases in temperature/water flows, changes in pH or dissolved oxygen) across a species range would arguably provide the most direct assessments of climate vulnerability, yet such information is exceptionally challenging to attain at a large scale, especially for a widespread species like kokanee. However, genomes carry signatures of past and current selection pressures and can provide insights into the evolutionary potential of populations and species to adapt to future climate change, as shown in an increasing number of studies, including here. The ongoing debate on the use of genome‐wide levels of standing genetic variation versus adaptive variation has mostly focused on their respective validity as proxies for adaptive potential in wild populations (Kardos et al., 2021; Teixeira & Huber, 2021). Here, we show that both standing and adaptive variation provide valuable information despite not always being concordant, offering complementary insights on the resilience of populations under climate change.

## CONFLICT OF INTEREST STATEMENT

The authors declare no competing interest.

## Supporting information


Data S1

## Data Availability

All genomic data are available through ENA/NCBI repositories and are linked to ENA project accession number PRJEB62788. All scripts and variant files are available at: http://doi.org/10.5281/zenodo.8349746 and http://doi.org/10.5281/zenodo.8349763, respectively.
