# Supplementary material for: Genomic vulnerability of a freshwater salmonid under climate change
Source: Evol Appl. 2023 Oct 27;17(2):e13602. doi: 10.1111/eva.13602 (PMC10853590; doi:10.1111/eva.13602)
Supplement: Supplementary file 1 — Data S1 [file EVA-17-e13602-s001.docx]

**Supplementary material**

**Figure S1.** Plot showing correlation coefficients of statistically significant correlations among all environmental variables considered.

**Table S1.** Information on population sampling locations, including coordinates, ecotype and sample size before (BF) and after (PF) filtering.

| Location | Coordinates  (longitude, latitude) | Ecotype | Sample size BF | Sample size PF | Sampling year |
| --- | --- | --- | --- | --- | --- |
| Anderson Lake | -122.3906179, 50.6541533 | Deep | 16 | 12 | 2021 |
| Arctic Lake | -121.6956189, 54.4295739 | Shore | 12 | 11 | 2019 |
| Arrow Lake (Lower-Mosquito) | -117.9608437, 50.1400347 | Stream | 10 | 8 | 2016 |
| Arrow Lake (Upper-Hill) | -117.8491607, 50.6725008 | Stream | 9 | 9 | 2015 |
| Bonaparte Lake | -120.5410622, 51.2647961 | Shore | 7 | 5 | 2021 |
| Christina Lake | -118.263629, 49.1458228 | Shore | 12 | 12 | 2011 |
| Cluculz Lake | -123.5528529, 53.8815497 | Shore | 6 | 4 | 2012 |
| Cowichan Lake | -124.2938747, 48.8882594 | Shore | 8 | 6 | 2021 |
| Dunn Lake | -120.1279726, 51.4274438 | Shore | 2 | 2 | 2021 |
| East Barriere Lake | -119.8026321, 51.271574 | Deep | 12 | 12 | 2019 |
| Kalamalka Lake | -119.3439333, 50.1751792 | Shore | 12 | 12 | 2018 |
| Kalamalka Lake | -119.2660313, 50.2288728 | Stream | 12 | 11 | 2018 |
| Kootenay Lake | -116.8074926, 49.4986685 | Stream | 12 | 9 | 2013 |
| Lac La Hache | -121.5769136, 51.8360334 | Shore | 4 | 3 | 2021 |
| Natelsby Lake | -121.7983454, 51.8995395 | Shore | 8 | 4 | 2018 |
| Nicola Lake | -120.5310579, 50.161573 | Stream | 9 | 9 | 2021 |
| Okanagan Lake | -119.4721881, 50.0078944 | Shore | 12 | 12 | 2017 |
| Okanagan Lake | -119.6911547, 49.7676906 | Stream | 12 | 12 | 2017 |
| Puntzi Lake | -124.0263194, 52.1977932 | Shore | 14 | 11 | 2021 |
| Shawnigan Lake | -123.6415078, 48.6360961 | Shore | 11 | 10 | 2021 |
| Sockeye Lake | -137.6257926, 60.5002411 | Shore | 16 | 10 | 2019 |
| Tchesinkut Lake | -125.642958, 54.0983352 | Shore | 12 | 11 | 2010 |
| Thutade Lake | -126.9441108, 56.8864649 | Shore | 10 | 8 | 2017 |
| Wood Lake | -119.4053864, 50.0640906 | Shore | 12 | 12 | 2021 |
| Wood Lake | -119.3882047, 50.0776396 | Stream | 13 | 9 | 2017 |

**Table S2.** Proportion of variation explained by all factors together (Full) or separately in the partial RDA (pRDA) based on either sequence or structural variation.

|  | Sequence variation | Structural variation |
| --- | --- | --- |
| Full | 18.5% | 25.3% |
| Climate | 6.3% | 7.3% |
| Population structure | 4% | 4.9% |
| Geography | 2.2% | 2.6% |
| Ecotype | 1.7% | 2.5% |

**Table S3.** Functional annotation and gene ontology terms (biological processes, KEGG pathways and zebrafish genotypes) significantly enriched across the strong sequence outliers.

| Database | **Enrichment FDR** | **Fold Enrichment** | **Pathway** | **Genes** |
| --- | --- | --- | --- | --- |
| Biological Processes | **0.00650506** | 1.82422415 | Anatomical structure morphogenesis | tnfrsf21  sema6d  epha7  tenm3  prdm5  fgfr1b  mybpc3  dcn  cdh13  cdh6  cdh4  rhobtb4  plxnb1b  acvrl1  flncb  parvb  nkd1  snx5  mcrs1  tmod4  fzd3a  erbb2  mapk1  dscamb  angpt1  epb41l5  itga9  ptprja  oxtr  pcdh19  brwd3  cyfip2  kif1b  nr2f2  rpgrip1l  ptger3  fdx1  ilk  gfpt1  tfap2a  bmp3  satb2  myo5b  wnt9a  nphp4  foxg1a  lama3  cntnap1  diaph2  espn  hspg2  sp5a  nlgn1  robo2  plxdc1  megf8  slitrk4  ppp1r16b  hmx1  sgcd  pygo1  pacsin3  gap43  col22a1 |
| Biological Processes | **0.01250413** | 1.85414954 | Nervous system development | tnfrsf21  sema6d  tenm1  tcf7l2  ntrk1  epha7  tenm3  pou3f1  msi1  cdh4  plxnb1b  smarcd1  sptan1  nkd1  snx5  fzd3a  erbb2  dscamb  cdk16  epb41l5  oxtr  lingo1a  pcdh19  cyfip2  kif1b  fstl5  rpgrip1l  tfap2a  dok7  clstn2  myo5b  wnt9a  astn1  btbd9  pou4f2  foxg1a  lama3  mgat5  olig3  cntnap1  asphd1  ralgapa1  nlgn1  brinp1  robo2  slitrk4  hmx1  pacsin3  akt1  gap43  zfhx3  CABZ01075572.1 |
| Biological Processes | **0.01250413** | 1.69888124 | Animal organ development | sema6d  col4a4  tcf7l2  prdm5  hsf1  pou3f1  mybpc3  snrpd1  dcn  ildr2  ddx4  cdh6  cdh4  pnn  acvrl1  flncb  smarcd1  nkd1  snx5  mcrs1  fzd3a  erbb2  pdlim5b  myf6  tox  epb41l5  oxtr  pcdh19  cyfip2  kif1b  nr2f2  cx43  rpgrip1l  zfand3  nog3  armc4  ctcf  ilk  gfpt1  tfap2a  dok7  bmp3  myo5b  scube1  wnt9a  btbd9  nphp4  glis3  pou4f2  sh2b3  foxg1a  furinb  lama3  mgat5  olig3  asphd1  hspg2  ralgapa1  setd5  megf8  hmx1  sgcd  pygo1  lsr  pacsin3  sf3a3  tfr1a  col22a1  zfhx3 |
| Biological Processes | **0.02998696** | 1.65204927 | Cellular developmental process | tnfrsf21  sema6d  tenm1  ntrk1  epha7  tenm3  fgfr1b  ddx4  cdh4  pnn  plxnb1b  elk3  flncb  smarcd1  parvb  sptan1  nkd1  snx5  tmod4  fzd3a  uvrag  erbb2  dscamb  cdk16  tox  nr1d1  lingo1a  dazl  cyfip2  kif1b  fstl5  nr2f2  fstl4  rpgrip1l  nog3  ilk  gfpt1  tfap2a  dok7  bmp3  myo5b  wnt9a  astn1  mettl5  glis3  zp3d.2  foxg1a  lama3  mgat5  olig3  cntnap1  espn  brinp1  robo2  slitrk4  ppp1r16b  hmx1  pum1  pacsin3  akt1  gap43  sf3a3  tfr1a  zfhx3  CABZ01075572.1 |
| Biological Processes | **0.04397419** | 27.8764253 | Mitochondrial RNA processing | elac2  trmt10a  prorp |
| Kegg pathways | **0.00083323** | 2.80870784 | MAPK signaling pathway | nr4a1  ntrk1  fgfr1b  ngfb  flncb  tab2  ppp3cb  erbb2  mapk1  rps6ka2  angpt1  rps6ka1  mapk11  dusp10  casp3b  fgfr2  map3k8  rasgrp2  ngfra  prkacaa  irak4  fgf3  cacna1da  atf4a |
| Kegg pathways | **0.00542959** | 2.64733383 | Calcium signaling pathway | ntrk1  adrb1  fgfr1b  itpr2  ngfb  adra1d  ppp3cb  erbb2  oxtr  ptafr  slc8a1b  ptger3  fgfr2  htr5ab  tnnc2  ryr3  prkacaa  tpcn2  fgf3  cacna1da |
| Kegg pathways | **0.01949287** | 2.09203942 | Neuroactive ligand-receptor interaction | crhr1  adrb1  gabra4  gabrp  adra1d  grik4  grm3  oxtr  s1pr2  gnrhr4  ptgir  grm4  ptafr  grid1b  gabrg2  nmbr  grid2  ptger3  rxfp3  htr5ab  insl5a  gabrb1  adra2b  GRIK2 |
| Kegg pathways | **0.01949287** | 3.3403124 | Cell adhesion molecules | ptprfb  ncam2  cdh2  cadm1a  itga9  nrxn3a  cntnap1  esama  nlgn1  vtcn1  jam3a |
| Phenotype ZFIN | **0.00420188** | 3.48455316 | Whole-organism viability abnormal | syf2  adrb1  msi1  snrpd1  acvrl1  slc6a9  cdh2  cyfip2  slc17a6b  ext2  gfpt1  dido1  rnf121  pole2  sf3a3  ccdc40  tfr1a  tbxta |
| Phenotype ZFIN | **0.00420188** | 2.68248896 | Whole-organism decreased-length abnormal | tcf7l2  wwox  msi1  pomgnt2  dcn  casr  cdh4  cdh2  mapk1  pcdh19  cyfip2  cx43  ppp1cb  rpgrip1l  ctcf  ext2  gfpt1  opa1  abhd12  diaph2  hspg2  klf4  zmiz2  tbxta  sec23a |
| Phenotype ZFIN | **0.00420188** | 7.43371341 | Post-vent-region curved-dorsal abnormal | cdh2  epb41l5  etfdh  rpgrip1l  ctcf  ext2  abhd12  hspg2 |
| Phenotype ZFIN | **0.01535133** | 3.51594553 | Whole-organism lethal-(sensu-genetics) abnormal | tcf7l2  slc6a9  cdh2  sptan1  cyfip2  s1pr2  kif1b  slc17a6b  ctcf  ext2  tfap2a  rnf121  ccdc40  tbxta |
| Phenotype ZFIN | **0.01621301** | 3.59517389 | Blood-circulation disrupted abnormal | acvrl1  cdh2  flncb  snx5  klf6a  ptprja  s1pr2  scube2  ilk  spns2  opa1  sgcd  ptpn12 |
| Phenotype ZFIN | **0.01621301** | 27.8764253 | Retinal-ganglion-cell irregular-spatial-pattern abnormal | cyfip2  slc17a6b  ext2 |
| Phenotype ZFIN | **0.01621301** | 15.4869029 | Brain disorganized abnormal | cdh4  cdh2  pcdh19  fmr1 |
| Phenotype ZFIN | **0.02390435** | 2.01728993 | Eye decreased-size abnormal | prdm5  wwox  hsf1  psmb1  msi1  pnpla6  cdh6  cdh4  znf503  cdh2  flncb  smarcd1  mcrs1  usp20  klf6a  ctcf  ilk  tfap2a  cep290  abhd11  opa1  abhd12  suds3  toe1  klf4  hmx1  ninl  pole2  sema6a  usp43a  inpp5b  agtpbp1 |
| Phenotype ZFIN | **0.02410607** | 2.01720164 | Pericardium edematous abnormal | ruvbl1  wwox  psmb1  msi1  pnpla6  mybpc3  mphosph10  cdh6  pnn  acvrl1  flncb  parvb  usp20  klf6a  s1pr2  fmr1  nr2f2  ilk  cep290  nphp4  opa1  suds3  myzap  hspg2  setd5  ninl  sgcd  akt1  ptpn12  usp43a  inpp5b |
| Phenotype ZFIN | **0.02410607** | 23.2303544 | Habenula process-quality abnormal | tcf7l2  ulk2  tbxta |
| Phenotype ZFIN | **0.03204904** | 6.4828896 | Surface-structure quality abnormal | cdh2  cx43  ext2  myo5b  edar  sec23a |
| Phenotype ZFIN | **0.03204904** | 19.9117323 | Retinal-ganglion-cell displaced abnormal | cyfip2  slc17a6b  ext2 |
| Phenotype ZFIN | **0.03500585** | 10.9319315 | Eye edematous abnormal | itga9  cx43  sall4  opa1 |
| Phenotype ZFIN | **0.03500585** | 7.74345146 | Meckel's-cartilage decreased-length abnormal | dcn  fmr1  ext2  tfap2a  spns2 |
| Phenotype ZFIN | **0.03756799** | 2.05942858 | Head decreased-size abnormal | ruvbl1  syf2  psmb1  msi1  pnpla6  pomgnt2  mphosph10  cdh6  megf10  cdh2  smarcd1  usp20  klf6a  etfdh  rpgrip1l  dvl2  ctcf  btbd9  abhd11  suds3  ralgapa1  toe1  setd5  pole2  usp43a |
| Phenotype ZFIN | **0.03756799** | 15.4869029 | Posterior-lateral-line-nerve decreased-amount abnormal | sptan1  erbb2  abhd12 |
| Phenotype ZFIN | **0.03756799** | 4.78272002 | Whole-organism increased-curvature abnormal | msi1  cdh6  jade1  cep290  nphp4  ninl  zmiz2 |
| Phenotype ZFIN | **0.03756799** | 6.63724411 | Cartilage-development disrupted abnormal | dcn  pnn  ext2  fgf3  sec23a |
| Phenotype ZFIN | **0.03756799** | 7.03950133 | Trunk decreased-amount abnormal | scube2  elavl4  tfap2a  tfec  rspo1 |
| Phenotype ZFIN | **0.03756799** | 10.324602 | Retinal-ganglion-cell-axon-guidance quality abnormal | cyfip2  slc17a6b  ext2  robo2 |
| Phenotype ZFIN | **0.03756799** | 9.29214176 | Retinal-ganglion-cell-axon-guidance process-quality abnormal | cyfip2  slc17a6b  ext2  robo2 |
| Phenotype ZFIN | **0.03756799** | 30.9738059 | Neural-rod disorganized abnormal | cdh2  pcdh19 |
| Phenotype ZFIN | **0.03756799** | 17.4227658 | Retina structure abnormal | hsf1  cdh6  cdh4 |
| Phenotype ZFIN | **0.03756799** | 30.9738059 | Trunk-musculature retracted abnormal | parvb  ilk |
| Phenotype ZFIN | **0.03756799** | 30.9738059 | Slow-muscle-cell process-quality abnormal | stac3  tpcn2 |
| Phenotype ZFIN | **0.03756799** | 30.9738059 | Rohon-Beard-neuron mislocalized-adaxially abnormal | ptprfb  ext2 |
| Phenotype ZFIN | **0.03756799** | 30.9738059 | Rohon-Beard-neuron subdermal abnormal | ptprfb  ext2 |
| Phenotype ZFIN | **0.03756799** | 30.9738059 | Sensory-neuron-axon-guidance disrupted abnormal | ptprfb  ext2 |
| Phenotype ZFIN | **0.03756799** | 30.9738059 | Meckel's-cartilage sloped-downward abnormal | ext2  tfap2a |
| Phenotype ZFIN | **0.03756799** | 30.9738059 | Optic-vesicle malformed abnormal | pcdh19  foxg1a |
| Phenotype ZFIN | **0.03756799** | 30.9738059 | Caudal-fin blistered abnormal | s1pr2  spns2 |
| Phenotype ZFIN | **0.03756799** | 30.9738059 | Trunk-musculature refractivity abnormal | six1b  hspg2 |
| Phenotype ZFIN | **0.03756799** | 30.9738059 | Endoderm-development process-quality abnormal | ildr2  lsr |
| Phenotype ZFIN | **0.03756799** | 30.9738059 | Pancreatic-B-cell mislocalised abnormal | ildr2  lsr |
| Phenotype ZFIN | **0.03756799** | 30.9738059 | Notochord-cell-differentiation process-quality abnormal | pacsin3  tbxta |
| Phenotype ZFIN | **0.03756799** | 30.9738059 | Mandibular-arch-skeleton size abnormal | s1pr2  spns2 |
| Phenotype ZFIN | **0.03756799** | 30.9738059 | Pharyngeal-ectoderm disorganized abnormal | s1pr2  spns2 |
| Phenotype ZFIN | **0.03756799** | 30.9738059 | Pharyngeal-ectoderm increased-area abnormal | s1pr2  spns2 |
| Phenotype ZFIN | **0.03756799** | 30.9738059 | Oral-ectoderm hypoplastic abnormal | s1pr2  spns2 |
| Phenotype ZFIN | **0.03756799** | 30.9738059 | Oral-ectoderm disorganized abnormal | s1pr2  spns2 |
| Phenotype ZFIN | **0.03756799** | 30.9738059 | Oral-ectoderm mislocalised-anteriorly abnormal | s1pr2  spns2 |
| Phenotype ZFIN | **0.03756799** | 30.9738059 | Median-fin blistered abnormal | s1pr2  spns2 |
| Phenotype ZFIN | **0.03756799** | 30.9738059 | Heart-tube increased-amount abnormal | s1pr2  spns2 |
| Phenotype ZFIN | **0.03863529** | 12.6711024 | Slow-muscle-cell morphology abnormal | gfpt1  stac3  tpcn2 |
| Phenotype ZFIN | **0.04646863** | 7.43371341 | Brain increased-occurrence abnormal | fmr1  dvl2  toe1  hmx1 |
| Phenotype ZFIN | **0.04646863** | 11.6151772 | Retinal-ganglion-cell mislocalised abnormal | tenm3  cyfip2  robo2 |
| Phenotype ZFIN | **0.04646863** | 7.43371341 | CaP-motoneuron decreased-length abnormal | plxna3  plxnb1b  chodl  fus |
| Phenotype ZFIN | **0.04646863** | 7.43371341 | Larval-locomotory-behavior process-quality abnormal | cyfip2  fus  setd5  cacna1da |
| Phenotype ZFIN | **0.04825318** | 10.721702 | Eye-photoreceptor-cell decreased-amount abnormal | cdh6  abhd12  ninl |
| Phenotype ZFIN | **0.04825318** | 7.14780135 | Fin decreased-size abnormal | cx43  ext2  myo5b  tbxta |
| Phenotype ZFIN | **0.04825318** | 10.721702 | Sinus-venosus increased-accumulation abnormal | ilk  myzap  sgcd |
| Phenotype ZFIN | **0.04825318** | 10.721702 | Intestine morphology abnormal | ildr2  myo5b  lsr |
| Phenotype ZFIN | **0.04825318** | 10.721702 | Ceratohyal-cartilage decreased-length abnormal | dcn  ext2  spns2 |

**Table S4.** Genomic offset estimates for each combination of genomic variant (sequence [SNPs] or structural [SVs]) and climate change scenario (RCP2.6 and RCP8.5).

| Sampling location | SNPs-RCP2.6 | SNPs-RCP8.5 | SVs-RCP2.6 | SVs-RCP8.5 |
| --- | --- | --- | --- | --- |
| Anderson Lake | 0.01602521 | 0.02261081 | 0.00488004 | 0.00687939 |
| Arctic Lake | 0.02156788 | 0.02486018 | 0.00656582 | 0.00766435 |
| Arrow Lake (Lower-Mosquito) | 0.01933659 | 0.02592219 | 0.00607461 | 0.00807395 |
| Arrow Lake (Upper-Hill) | 0.0184196 | 0.02500519 | 0.00609669 | 0.00809604 |
| Bonaparte Lake | 0.0187516 | 0.02451399 | 0.00522713 | 0.00697656 |
| Christina Lake | 0.02420841 | 0.0305196 | 0.00746415 | 0.00938019 |
| Cluculz Lake | 0.01943277 | 0.02546956 | 0.00604622 | 0.00787895 |
| Cowichan Lake | 0.01629962 | 0.02206201 | 0.00496335 | 0.00671277 |
| Dunn Lake | 0.01542917 | 0.01999808 | 0.00428299 | 0.00569421 |
| East Barriere lake | 0.0175964 | 0.02418198 | 0.00584678 | 0.00784612 |
| Kalamalka Lake | 0.01881571 | 0.0251269 | 0.00598203 | 0.00789807 |
| Kootenay Lake | 0.02109602 | 0.02768161 | 0.00644957 | 0.00844892 |
| Lac La Hache | 0.02039051 | 0.02670171 | 0.00651888 | 0.00843492 |
| Natelsby Lake | 0.01634611 | 0.0226573 | 0.00523228 | 0.00714831 |
| Nicola Lake | 0.02124484 | 0.02755603 | 0.00701028 | 0.00892631 |
| Okanagan Lake | 0.01948238 | 0.02579357 | 0.00591472 | 0.00783076 |
| Puntzi Lake | 0.01908821 | 0.025125 | 0.00595843 | 0.00779116 |
| Shawnigan Lake | 0.02098988 | 0.02565468 | 0.00605037 | 0.00746657 |
| Sockeye Lake | 0.00709537 | 0.01417608 | 0.00185876 | 0.0047143 |
| Tchesinkut Lake | 0.02477611 | 0.02725579 | 0.00748488 | 0.00830649 |
| Thutade Lake | 0.01453157 | 0.01971539 | 0.00440714 | 0.00572585 |
| Wood Lake | 0.02084451 | 0.0274301 | 0.00638558 | 0.00838492 |
